# Hydroxypropyl-***β***-cyclodextrin: A Novel Transungual Permeation Enhancer for Development of Topical Drug Delivery System for Onychomycosis

**DOI:** 10.1155/2014/950358

**Published:** 2014-08-06

**Authors:** Pradeep Chouhan, T. R. Saini

**Affiliations:** Industrial Pharmacy Research Lab, Department of Pharmacy, Shri G. S. Institute of Technology and Science, 23 Park Road, Indore, Madhya Pradesh 452003, India

## Abstract

The treatment of onychomycosis is a challenging task because of unique barrier properties of the nail plate which hampers the passage of antifungal drugs in a concentration required to eradicate the deeply seated causative fungi in the nail bed. In present investigation, application of hydroxypropyl-*β*-cyclodextrin (HP-*β*-CD) was established as an effective and nail friendly transungual drug permeation enhancer especially for poorly water soluble drugs using terbinafine hydrochloride as a poorly soluble drug. HP-*β*-CD significantly improves hydration of nail plates and increases solubility of terbinafine hydrochloride in the aqueous environment available therein, which leads to uninterrupted drug permeation through water filled pores of hydrogel-like structure of hydrated nail plates. A nail lacquer formulation was designed with an objective to deliver the drug in an effective concentration across nail plates, using HP-*β*-CD as a permeation enhancer. The formulations containing HP-*β*-CD showed higher flux than the control formulation in in vitro drug permeation study. The formulation containing 10% w/v of HP-*β*-CD showed maximum flux of 4.586 ± 0.08 *μ*g/mL/cm^2^ as compared to the control flux of 0.868 ± 0.06 *μ*g/mL/cm^2^. This finding supports application of HP-*β*-CD as an effective permeation enhancer for transungual delivery of terbinafine hydrochloride and possibly other poorly water soluble drugs where HP-*β*-CD can act as a solubilizer.

## 1. Introduction

Onychomycosis is the most common nail fungal disease, which causes discoloration, thickening, hardening, and crumbling of the infected nails [1].

The delivery and maintenance of an effective concentration of antimycotic drugs higher than their minimum inhibitory concentration (MIC) across nail plate are a major challenge faced in the treatment of onychomycosis. The conventional drug therapy involves daily administration of antifungal drugs through oral and topical routes. In general, the oral antifungal therapy is associated with severe systemic and gastrointestinal side effects. Terbinafine hydrochloride has been particularly reported to cause hepatotoxicity [2–4]; thus, a routine liver function test is recommended for patients taking continuous treatment of terbinafine hydrochloride for more than one month [1]. To eliminate its systemic toxicity, topical route of drug administration could be used in place of oral route. The inherent problem with transungual formulations is their poor drug permeability through nail plate and, therefore, the drug flux is mostly lower than its MIC [5, 6]. Nail lacquer formulations have, however, emerged as an effective topical drug delivery system for treating nail fungal diseases [1, 7]. Nonetheless, as antifungal drugs are mostly water insoluble and show poor transungual permeability, their delivery across the nail plate in adequate concentration from nail lacquer formulation is not possible.

The main cause of poor transungual permeation of these drugs is impermeable nature of the keratinized nail plate and entrapment of drugs in nail keratin during their passage. The drug transport across nail plate is principally influenced by thickness and hydration of nail plate, physicochemical properties of drug molecule (molecular size, ionization, and partition coefficient), and formulation characteristics (nature of vehicle and drug concentration). Recent findings have revealed that drug permeation across nail plate can be significantly enhanced in presence of keratinolytic agents which can disrupt keratin network of nail plate and thus weaken its barrier property which results in improvement of drug permeation [8–10]. The keratinolytic agents, for example, urea and salicylic acid, destabilize hydrogen bonds and sulfhydryl compounds; for example, n-acetyl-l-cysteine cleaves the disulfide linkages in nail keratin and alters the permeability of nail plates [1]. As the above permeation enhancers irreversibly damage the microstructure of nail plate and so its functioning, therefore, such types of permeation enhancers are not considered as safe and nail friendly. Therefore, a continuous search for other permeation enhancement mechanisms and/or application of other permeation enhancers which do not damage integrity of nail plate is still on.

The nail also contains 10–30% water, which not only imparts flexibility to it but also controls its barrier property for drug molecules [7]. The nail swells as water is absorbed and the hydrated nail behaves like a hydrogel with a dense network of watery pores through which molecules can permeate [8]. The hydrated nail could, thus, allow permeation of small water soluble molecules which was otherwise not possible. The rate of permeation of such drug molecules would be then principally influenced by their water solubility [11]. If the drug is water soluble, its permeation would not be hindered, but if it is water insoluble, its permeation would be limited by its water solubility and dissolution rate. Working on this hypothesis, it was theorized that the substance like hydroxypropyl-*β*-cyclodextrin (HP-*β*-CD) which could increase hydration of nail plate as well as aqueous solubility of drugs in aqueous microenvironment under the nail lacquer film would also facilitate its transungual permeation. In the present investigation above concept was explored for transungual permeation enhancement of a model of poorly water soluble antifungal drug, terbinafine hydrochloride (log *p* = 5.9), using HP-*β*-CD as permeation enhancer. A nail lacquer drug delivery system was designed and evaluated for transungual drug delivery of terbinafine hydrochloride across the nail plate through topical route.

Terbinafine hydrochloride, a potent antifungal agent from allylamine antifungals, was selected as a model drug because it is very effective in treatment of dermatophyte infections and is a drug of choice for treatment of onychomycosis.

## 2. Materials and Methods

### 2.1. Materials

Terbinafine hydrochloride was received as a kind gift from M/s Elder Pharmaceuticals Ltd., Mumbai, India, and cellulose acetate was obtained as a free sample from M/s Torrent Pharmaceuticals Ltd., Ahmadabad, India. Triethyl citrate was received as a free sample from Morflex, Inc., North Carolina, USA, and HP-*β*-CD was obtained from Signet Chemical Corporation Pvt. Ltd., Mumbai, India. Analytical grade isopropyl alcohol and acetone and HPLC grade water, triethylamine, acetic acid, and methanol were purchased from Merck, India. All solutions were prepared in deionized water.

#### 2.1.1. Nail Clippings

The nail clippings were collected from healthy human volunteers (male and female, age 25–50). The nail clippings were washed three times with 70% v/v ethanol followed by rinsing with deionized water. They were dried overnight in an open petri-dish at room temperature. The dried nail clippings were either used immediately or stored at controlled temperature (2–8°C) in aluminum capped vials [12]. The nail clippings were cut into pieces of 10 × 10 mm size and characterized for average thickness and weight. The thickness of each nail clipping was measured with a digital caliper (Micrometer MI-1000, Mitutoyo, Japan) at three different points. Thickness of nail clippings was found to be in the range of 0.4-0.5 mm. Similarly, each nail clipping was weighed on an analytical balance (AUX220, Shimadzu, Japan), and the average weight of the clippings was found to be 41.77 ± 2.70 mg.

### 2.2. Methods 

#### 2.2.1. Nail Hydration Enhancement Study

To evaluate the nail hydration enhancement property of HP-*β*-CD, nail clippings were weighed and placed in glass vials filled with 1%, 2%, 5%, and 10% w/v aqueous solutions of HP-*β*-CD. Simultaneously nail clipping in deionized water (0% w/v HP-*β*-CD) was used as test control. The glass vials were sealed and allowed to swell for 24 hr at room temperature; they were then removed and wiped with tissue paper and weighed [13].

#### 2.2.2. Solubility Study

To determine the effect of HP-*β*-CD on solubility of terbinafine hydrochloride, an excess amount of the drug was added to 0, 2, 4, 6, 8, and 10% w/v aqueous solution of HP-*β*-CD. The samples were kept in a shaking water bath maintained at 32°C for a period of 24 hr. The samples were immediately filtered through 0.45 *μ*m membrane filter and after suitable dilution with ethanol analyzed at 283 nm using UV/visible spectrophotometer (UV1700 Pharmaspec, Shimadzu, Japan).

#### 2.2.3. Preparation of Nail Lacquer Drug Delivery Formulation

A nail lacquer drug delivery formulation of terbinafine hydrochloride (formulation code L0) was designed using cellulose acetate and ethyl cellulose as film forming polymers, triethyl citrate as plasticizer, and isopropyl alcohol and acetone as solvents (Table 1).

The nail lacquer was prepared by adding cellulose acetate in acetone and ethyl cellulose in isopropyl alcohol under continuous stirring. Both the solutions were mixed together and terbinafine hydrochloride and triethyl citrate were added to it under stirring till a clear solution was formed. The measured amount of deionized water was added to the resulting solution and mixed well and stored in an air tight container till used.

#### 2.2.4. In Vitro Transungual Permeation Study

In order to determine the transungual permeation of terbinafine hydrochloride from nail lacquer formulation (formulation code L0), an in vitro drug permeation study was carried out for 48 hr using the Franz diffusion cell apparatus. Nail lacquer formulation equal to 200 *μ*g drug was applied on nail clippings. After formation of a dry film, nail clippings were placed in the nail adapters customized to hold the nail plates and sandwiched between receptor and donor compartments of diffusion cell. The nail adapter has 8 mm diameter hole, so the effective diffusion area of the nail plates was equal to 0.502 cm^2^. Receptor compartment was filled with 13 mL phosphate buffer, pH 7.0 (containing 0.01% w/v sodium azide as a microbial growth inhibitor and 0.1% w/v thiourea as an antioxidant). The drug permeation medium was stirred by externally driven, Teflon coated small magnetic bead.

Temperature of the receptor compartment was maintained at 32 ± 0.5°C by circulation of thermostatic water across the cells [14]. The diffusion cells were covered with aluminum foil to avoid contamination and evaporation of drug permeation medium. Expectedly, the rate of transungual drug permeation was excessively low and drug permeated was below the detection limit of presently used HPLC method. A new sampling method was, therefore, developed. For each study 4 sampling time points were fixed and a dedicated Franz diffusion cell was reserved for sampling at each sampling point. At each sampling point, whole 13 mL fluid of receptor compartment of one Franz diffusion cell was collected. Extraction of terbinafine hydrochloride was done from the collected fluid by evaporating it using a rotary vacuum evaporator (R205, Buchi, Switzerland). After complete evaporation, 3 mL methanol was added to it and sonicated for 2 min in a bath sonicator. The resulting methanolic solution was filtered and analyzed for drug content by the HPLC method.

#### 2.2.5. Analytical Method

**Figure 1:**
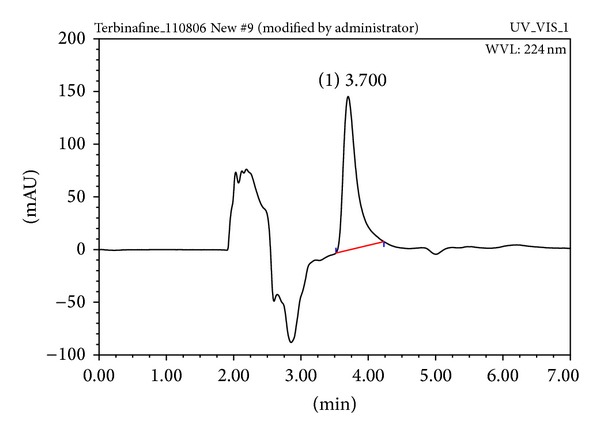
A representative chromatogram of terbinafine hydrochloride in drug permeation medium.

Terbinafine hydrochloride in test samples was analyzed on a Dionex HPLC system (P680 HPLC, Dionex, USA) using Hypersil C18 BDS analytical column (4.6 mm × 250 mm, 5.0 *μ*m) and an aqueous solution of triethyl amine (0.1%) and methanol (60 : 40) adjusted to pH 6 with acetic acid as the mobile phase. The elution flow rate was maintained at 1 mL/min and the injection volume 20 *μ*L and column effluent were monitored at 224 nm. The method was validated by determining linearity, precision, and accuracy. The range of calibration curve was found to be 1–10 *μ*g/mL (*R* ^2^ = 0.999). The representative chromatogram of terbinafine hydrochloride in drug permeation medium is shown in Figure 1; retention time of the peak was obtained at 3.7 ± 0.12 min (*n* = 6).

#### 2.2.6. Evaluation of Nail Lacquer Film Characteristics

*Drug Content*. One mL nail lacquer formulation was diluted up to 100 mL with methanol. One mL of this solution was diluted up to 10 mL with methanol-water combination (95 : 5); the resultant solution was filtered through 0.22 *μ*m membrane filter and analyzed by HPLC method.

*Film Thickness*. One mL of formulated nail lacquer was taken in 8 cm diameter petri-dish and was spread evenly with a lacquer applicator brush. The nail lacquer was allowed to dry at room temperature. After complete drying the film was separated from the petri-dish. The average film thickness was calculated by measuring the thickness of the film at three different places using a digital caliper (Micrometer MI-1000, Mitutoyo, Japan).

*Nonvolatile Content*. One gram sample of each formulated nail lacquer was spread out evenly in a glass petri-dish of about 8 cm diameter. Petri-dishes were weighed and dried at 105°C for 1 hr. The difference in weight of petri-dishes before and after drying was determined and reported as weight of nonvolatile contents in nail lacquer.

*Film Drying Time and Glossiness*. A film of formulated nail lacquers was casted on a glass petri-dish with the help of a lacquer applicator brush. The time to form a “dry to touch” film was noted as drying time for respective film. The glossiness of the prepared film was determined by visual inspection. The intensity of glossiness was measured in three different grades, that is, good (+ +), very good (+ + +), and excellent (+ + + +).

#### 2.2.7. Effect of HP-*β*-CD on Transungual Permeation of Terbinafine Hydrochloride

In the nail hydration enhancement study, it was observed that when used in concentrations above 5% w/v, HP-*β*-CD significantly increased hydration of nail clippings (hydration enhancement factor, HEF_24_) (Table 3). The HEF_24_ (1) of transungual permeation enhancers has a direct relationship with their transungual drug permeation enhancement efficiency [13]. Therefore, above 5% w/v HP-*β*-CD was considered as its minimum effective permeation enhancer concentration and hence the formulation containing 6% w/v HP-*β*-CD (formulation code L6) was designed to confirm the effect of HP-*β*-CD on transungual permeation of terbinafine hydrochloride.

The nail lacquer drug delivery formulation containing 6% w/v HP-*β*-CD was prepared (Table 2) by procedure mentioned earlier for nail lacquer drug delivery formulation code L0. The measured amount of HP-*β*-CD was dissolved separately in deionized water and added to other ingredients of nail lacquer.

#### 2.2.8. Optimization of HP-*β*-CD Concentration as Transungual Permeation Enhancer

To optimize HP-*β*-CD concentration as a transungual permeation enhancer, 4 more nail lacquer drug delivery formulations containing 7%, 8%, 9%, and 10% w/v HP-*β*-CD (formulation codes L7 to L10, resp., Table 2) were designed and evaluated.

## 3. Calculation and Data Analysis

The hydration enhancement factor, HEF_24_ (1), of nail clippings was determined for each concentration of HP-*β*-CD by method reported earlier [13]. Consider

(1) $$\begin{array}{c} {\text{HEF}}_{\text{24}}=\frac{\text{Wp}}{\text{Wc}}, \end{array}$$

where

 HEF_24_ = hydration enhancement factor calculated in 24 hr;
 Wp = % weight gain of nail clipping exposed to permeation enhancer solution;
 Wc = % weight gain of nail clipping exposed to deionized water (control).

The cumulative amount of drug permeated through the nail plate (*Q*) in the in vitro drug permeation study was plotted against time (*t*), and the steady state flux (2) of permeated drug (*J*) was calculated from the slope of the linear portion of the plot [8]. Consider

(2) $$\begin{array}{c} J=\frac{\Delta Q}{A\Delta t}, \end{array}$$

where

 
*J* = flux;
 
*A* = diffusion area;
 Δ*Q*/Δ*t* = slope of the cumulative amount of drug permeated against time plot.

Statistical correlation between flux of permeated drug and concentration of HP-*β*-CD in nail lacquer formulation was determined using GraphPad Prism-6.04 software (GraphPad Software, Inc.).

## 4. Result and Discussion

The human nail plate is a highly ordered structure, chemically comprised of sulfur-rich *α*-keratins (~80%), water (10–30%), and lipids (0.1–1.0%) [8]. The nail plate contains about 10.5% cysteine which holds keratin fibers together and maintains the structural integrity and mechanical stability of nail [15]. The nail plates are resistant to external influences but they change their physical properties when soaked in water. The hydrated nail plates become softer with increased flexibility. The hydrated nail behaves like a hydrogel with a network of water filled pores through which more drug molecules can permeate [1].

**Figure 2:**
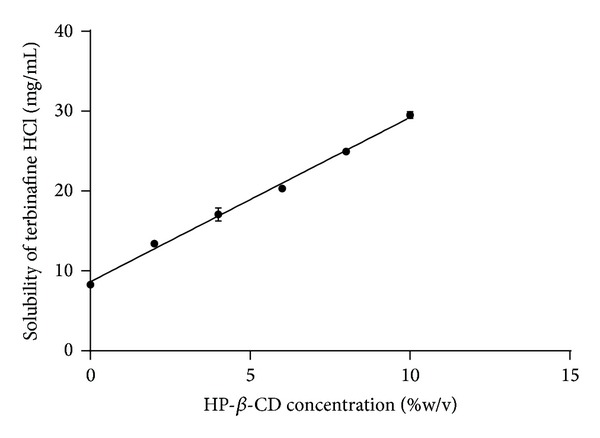
Solubility of terbinafine hydrochloride in the presence of HP-*β*-CD. Values are expressed as mean ± S.D. (*n* = 3).

Solubility study was performed at 32°C to determine the effect of HP-*β*-CD on solubility of terbinafine hydrochloride at condition of ex vivo permeation study. Solubility of the drug was increased as a function of HP-*β*-CD concentration.  Figure 2 shows that the solubility of terbinafine hydrochloride is linearly increasing with the concentration of HP-*β*-CD in water.

The water absorption property of the nail plate is also influenced by some chemical agents. The hydration enhancement property of such agents is, therefore, used as a preformulation screening tool to evaluate their application as drug permeation enhancer, in development of transungual formulations [13]. The application of HP-*β*-CD as a permeation enhancer in the present study was evaluated by hydration enhancement factor (HEF_24_). The nail clippings were exposed for 24 hr to different concentrations, that is, 0%, 1%, 2%, 5%, and 10% w/v of HP-*β*-CD in water. Nail clippings immersed in 10% w/v HP-*β*-CD solution showed maximum HEF_24_, that is, 1.23 ± 0.17, compared with nail clippings soaked in 1% (1.04 ± 0.28), 2% (1.04 ± 0.03), and 5% w/v (1.15 ± 0.13) HP-*β*-CD solution (Table 3). These results showed that the HP-*β*-CD can significantly improve hydration of nail clippings in more than 5% w/v concentration; therefore, the concentration above 5% w/v HP-*β*-CD was considered as permeation enhancer concentration in further experimentations.

Minimum inhibitory concentration (MIC) of terbinafine hydrochloride for most of the onychomycotic fungi is 1.0 *μ*g/mL [16]. As the drug permeation flux without permeation enhancer (formulation code L0, Table 1) was 0.868 ± 0.06 *μ*g/mL/cm^2^ only (Table 4) which was lower than the MIC of terbinafine hydrochloride, the drug delivery across nail plate above 1.0 *μ*g/mL concentration by topical route with the help of HP-*β*-CD as a permeation enhancer was mainly targeted in present study. Firstly, a nail lacquer formulation containing 6% w/v HP-*β*-CD was designed (formulation code L6, Table 2) which showed significantly higher flux across nail plate, that is, 3.908 ± 0.05 *μ*g/mL/cm^2^ (Table 4). It is in agreement with the findings of hydration study and confirmed the utility of HP-*β*-CD as a transungual permeation enhancer in nail lacquer formulation.

Further, to optimize concentration of HP-*β*-CD as permeation enhancer, four more batches of nail lacquer formulations were designed containing 7, 8, 9, and 10% w/v HP-*β*-CD (Table 2). More than 10% w/v HP-*β*-CD could not be used because of its solubility limitation in present formulation.  Table 5 shows evaluation parameters of prepared nail lacquer formulations. For all nail lacquer formulations, film drying time was found to be between 60 and 70 seconds. After drying, a dried film of 0.2 ± 0.03 mm thickness was formed. The nonvolatile content of nail lacquer formulations was found to be in the range of 150 to 170 mg/gram of the formulation; these values showed uniformity of nonvolatile contents of all nail lacquer formulations. Drug content of all nail lacquer formulations was found to be consistent and well within limits.

**Figure 3:**
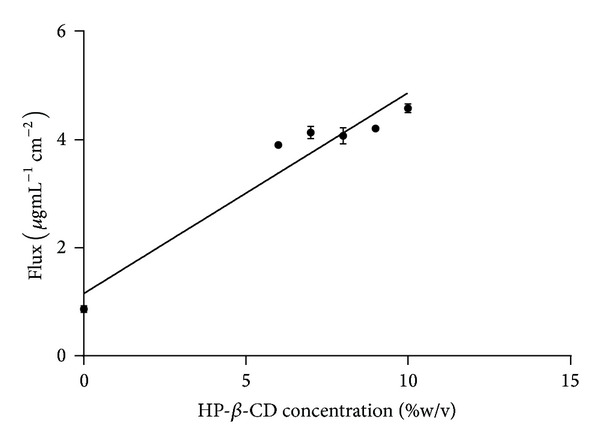
Nonlinear curve fitting between HP-*β*-CD concentrations and their effect on drug flux across nail clippings (*R* ^2^ = 0.929).

All four nail lacquer drug delivery formulations (formulation codes L7–L10) showed significantly higher drug permeation across nail plates in 48 hr, compared with the control formulation (formulation code L0). The drug permeation flux across nail clippings was progressively increased with increasing concentration of HP-*β*-CD in nail lacquer drug delivery formulations. To support above statement statistical analysis of the data was performed using GraphPad Prism-6.04 software (GraphPad Software, Inc.). A nonlinear regression curve fitting was performed to determine correlation between HP-*β*-CD concentrations in nail lacquer formulations and their effect on obtained flux values. As shown in Figure 3, a good correlation was observed between HP-*β*-CD concentration and obtained flux values as confirmed by correlation coefficient (*R* ^2^ = 0.929).

The formulation containing 10% w/v of HP-*β*-CD showed maximum flux, that is, 4.586 ± 0.08 *μ*g/mL/cm^2^ (Table 4). Permeated concentration of the drug across nail clippings from above formulation was much higher than the MIC of terbinafine hydrochloride for onychomycotic fungi.

## 5. Conclusion

The developed nail lacquer drug delivery formulation of terbinafine hydrochloride using 10% w/v HP-*β*-CD as a permeation enhancer (formulation code L10) could successfully deliver the desired quantity of terbinafine hydrochloride through topical route and thus can be considered as an effective drug delivery system for topical therapy of onychomycosis. The HP-*β*-CD showed enhanced permeation of terbinafine hydrochloride across the nail plate by virtue of increasing nail hydration ability as well as aqueous solubility of the drug without damaging the nail plate integrity and therefore establishes its applicability as a nail friendly transungual drug permeation enhancer for poorly water soluble drugs.

## Figures and Tables

**Table 1:** Composition of nail lacquer drug delivery formulation of terbinafine hydrochloride (formulation code L0).

Ingredient	Concentration (% w/v)
Terbinafine hydrochloride	1.0
Cellulose acetate	2.0
Triethyl citrate	0.1
Ethyl cellulose	3.0
Isopropyl alcohol	30.0
Acetone	48.9
Deionized water	15.0

**Table 2:** Composition of nail lacquer drug delivery formulations of terbinafine hydrochloride for optimization of HP-*β*-CD concentration.

Ingredient	Concentration (% w/v)				
	Formulation code				
	L6	L7	L8	L9	L10
Terbinafine hydrochloride	1	1	1	1	1
Cellulose acetate	2	2	2	2	2
Triethyl citrate	0.1	0.1	0.1	0.1	0.1
Ethyl cellulose	3	3	3	3	3
Isopropyl alcohol	30	30	30	30	30
Acetone	42.9	41.9	40.9	39.9	38.9
Deionized water	15	15	15	15	15
HP-*β*-CD	6	7	8	9	10

**Table 3:** Effect of HP-*β*-CD on hydration enhancement factor, HEF_24_.

S. number	Concentration of HP-*β*-CD (% w/v)	Hydration enhancement factor, HEF_24_ (mean ± S.D.) (*n* = 3)
1.	0	1.00 ± 0.02
2.	1	1.04 ± 0.28
3.	2	1.04 ± 0.03
4.	5	1.15 ± 0.13
5.	10	1.23 ± 0.17

**Table 4:** Effect of concentration of HP-*β*-CD on in vitro drug permeation (flux, *J*) of terbinafine hydrochloride from nail lacquer drug delivery formulations across nail clippings in 48 hr.

Concentration of HP-*β*-CD (% w/v)	Formulation code	Flux (*µ*g/mL/cm^2^) (mean ± S.D.) (*n* = 3)
0	L0	0.868 ± 0.06
6	L6	3.908 ± 0.05
7	L7	4.140 ± 0.11
8	L8	4.076 ± 0.15
9	L9	4.214 ± 0.05
10	L10	4.586 ± 0.08

**Table 5:** Evaluation of developed nail lacquer drug delivery formulations of terbinafine hydrochloride.

S. number	Formulation code	Drug content (%) (mean ± S.D.) (*n* = 3)	Thickness (mm) (mean ± S.D.) (*n* = 3)	Drying time (sec) (mean ± S.D.) (*n* = 3)	Film glossiness∗	Nonvolatile content (mg) (mean ± S.D.) (*n* = 3)
1	L0	100.13 ± 2.2	0.2 ± 0.03	60 ± 5	++++	150 ± 3.0
2	L6	98.89 ± 3.1	0.2 ± 0.03	70 ± 5	++++	160 ± 2.1
3	L7	100.93 ± 5.0	0.2 ± 0.03	65 ± 5	+++	166 ± 3.2
4	L8	101.10 ± 3.3	0.2 ± 0.02	70 ± 5	++++	162 ± 2.3
5	L9	99.87 ± 3.5	0.2 ± 0.02	65 ± 5	+++	165 ± 4.2
6	L10	100.89 ± 2.5	0.2 ± 0.03	70 ± 5	+++	170 ± 3.2

*++: good; +++: very good; ++++: excellent.
